# Assessment of a couples HIV counseling and testing program for pregnant women and their partners in antenatal care (ANC) in 7 provinces, Thailand

**DOI:** 10.1186/s12914-014-0039-2

**Published:** 2014-12-24

**Authors:** Rangsima Lolekha, Nareeluck Kullerk, Mitchell I Wolfe, Kanyarat Klumthanom, Thapanaporn Singhagowin, Sarika Pattanasin, Potjaman Sombat, Thananda Naiwatanakul, Chailai Leartvanangkul, Nipunporn Voramongkol

**Affiliations:** Global AIDS Program, Thailand MOPH—U.S. CDC Collaboration (TUC), Mail: P.O. Box 139, Nonthaburi, 11000 Thailand; Department of Health (DOH), Ministry of Public Health, Nonthaburi, Thailand; U.S. Centers for Disease Control and Prevention, Atlanta, GA USA

**Keywords:** Couples counseling, Pregnant women, ANC, Thailand

## Abstract

**Background:**

Couples HIV testing and counseling (CHTC) at antenatal care (ANC) settings allows pregnant women to learn the HIV status of themselves and their partners. Couples can make decisions together to prevent HIV transmission. In Thailand, men were tested at ANC settings only if their pregnant partners were HIV positive. A CHTC program based in ANC settings was developed and implemented at 16 pilot hospitals in 7 provinces during 2009–2010.

**Methods:**

Cross-sectional data were collected using standard data collection forms from all pregnant women and accompanying partners who presented at first ANC visit at 16 hospitals. CHTC data for women and partners were analyzed to determine service uptake and HIV test results among couples. In-depth interviews were conducted among hospital staff of participating hospitals during field supervision visits to assess feasibility and acceptability of CHTC services.

**Results:**

During October 2009-April 2010, 4,524 women initiating ANC were enrolled. Of these, 2,435 (54%) women came for ANC alone; 2,089 (46%) came with partners. Among men presenting with partners, 2,003 (96%) received couples counseling. Of these, 1,723 (86%) men and all pregnant women accepted HIV testing. Among 1,723 couples testing for HIV, 1,604 (93%) returned for test results. Of these, 1,567 (98%) were concordant negative, 6 (0.4%) were concordant positive and 17 (1%) were HIV discordant (7 male+/female- and 10 male-/female+). Nine of ten (90%) executive hospital staff reported high acceptability of CHTC services.

**Conclusions:**

CHTC implemented in ANC settings helps identify more HIV-positive men whose partners were negative than previous practice, with high acceptability among hospital staff.

## Background

In 2012, the World Health Organization (WHO) recommended that couples and partners of pregnant women in antenatal care (ANC) settings should be offered voluntary counseling and testing (VCT) with support for mutual disclosure [1,2], and also that antiretroviral therapy (ART) should be offered to HIV positive partners in serodiscordant relationships regardless of CD4 status [1] in order to reduce HIV transmission to uninfected partners [3]. Offering couples HIV testing and counseling (CHTC) at ANC settings provides many benefits including: increasing male participation in ANC services, enhancing communication between couples about safe sex practices [4,5], encouraging men to get tested and to know their HIV status, and preventing new HIV infections [1]. Couples who are aware of their partner’s and their own HIV status are more likely to adopt safe sex behaviors than people who are unaware of their HIV status [6,7]. If one or both members of a couple test positive, they can access and adhere to HIV treatment and care and interventions for prevention of mother-to-child HIV transmission (PMTCT). HIV-uninfected pregnant women and partners also receive benefits from CHTC through better-informed decisions about HIV prevention and reproductive health including contraception [1]. Offering CHTC at ANC settings may mitigate CHTC barriers and stigma discrimination because CHTC can be integrated into other routine maternal child health services and male participation activities routinely provided in ANC settings [8]. However, CHTC can be complex to implement because of limited number of staff at service delivery sites, acceptability problems among health care providers and clients, and potential adverse family consequences including conflict, separation, and intimate partner violence. Most reports from CHTC experiences are from projects based in low-income countries, particularly in sub-Saharan Africa [9,10], and Cambodia [11]; none exist for Thailand.

Thailand, an upper middle-income country, has a concentrated HIV epidemic with prevalence of 1.1% of the adult population [12]. Approximately 800,000 pregnant women deliver in Thailand each year, and 98% of pregnant women in Thailand receive ANC at health facilities, where HIV testing is routine and nearly all accept HIV testing [13]. In most ANC settings in Thailand, VCT is provided to men only when their pregnant partners are HIV positive. Studies in Thailand have reported high (30-50%) serodiscordant rates among HIV-infected couples [14]; about 32% of new HIV infections in 2012 were among low-risk co-habitating couples (e.g., husband to wife and wife to husband) [15]. One study in Thailand reported that 0.05% of pregnant women had HIV seroconversion during pregnancy (presumably from their HIV positive partners) [16]. Although Thailand has implemented a successful PMTCT program, the 2008 national PMTCT program evaluation [17] reported that only half of the partners of HIV-infected pregnant women received HIV testing within 6 months after delivery. In addition, only 15% of HIV-infected women had received CHTC at ANC settings, but more than 70% were interested in receiving CHTC [17] for the opportunity to more openly communicate with their partners about HIV and PMTCT during pregnancy and the postpartum period [17]. These data highlight the need for improved HIV testing rates in couples at ANC settings in Thailand.

In this paper, we describe the pilot implementation of a CHTC program in ANC settings in 17 hospitals in 7 provinces in Thailand during 2009–2010. Following this pilot implementation, Thailand national PMTCT guidelines 2010 [18,19] recommended routine CHTC in ANC clinics at public hospitals. Lessons learned from this pilot program provide recommendations for improvement and scale-up of this important program.

## Methods

### CHTC training and implementation in ANC setting

The Thai Ministry of Public Health (MOPH) and Thailand MOPH-U.S. Centers for Disease Control and Prevention Collaboration (TUC) developed a pilot project in 2008 in order to assess the feasibility and acceptability of CHTC implementation in Thai ANC settings. A training manual [20] was developed in Thai, adapted from the U.S. Centers for Disease Control and Prevention (CDC) CHTC manual [21]. In February 2009, a 4-day CHTC training for ANC settings was conducted by trainers from the MOPH Department of Health (DOH), TUC, and counselors from tertiary care hospitals, for 32 service providers (ANC nurses and counselors who provided individual VCT for pregnant women) comprising 17 hospitals in 7 provinces. Key contents of the training included the importance of CHTC in ANC services, psychosocial issues relating to couples relationships, counselor self-awareness, counseling skills required to work effectively with couples, guidance on providing pre-test and post-test CHTC (for HIV-seroconcordant negative, seroconcordant positive, and serodiscordant results), and administrative management to set up couples counseling service systems. Training methods included didactic sessions, role play, video demonstrations, small group discussions, and lectures. Following the training, trainees returned to their hospitals, trained their ANC teams, and implemented CHTC services as part of routine ANC services.

### Study population and procedures

**Figure 1:**
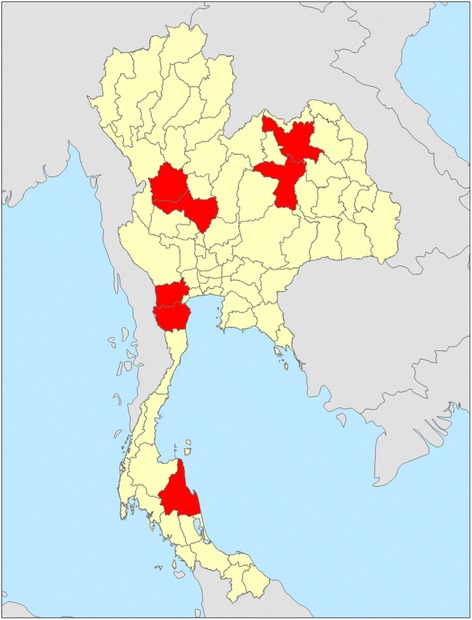
**Geographic area of seven provinces participated in CHTC project.**

From October 2009 to April 2010, all pregnant women and their partners at 17 hospitals in 7 provinces in the North, Northeastern, Central, and Southern regions of Thailand (Figure 1) were offered routine ANC and CHTC services, and were asked to consent to HIV testing at first ANC visit by counselors or nurses. A convenience sample of participants was recruited based on availability of staff. We did not collect data on the total number of pregnant women, their partners, or the number who were approached to participate at first ANC visit. Enrollment targeted 30 HIV-positive persons among those receiving CHTC services. We anticipated that enrollment of 30 HIV-positive persons would enable us to enroll at least 10 serodiscordant couples, including HIV-positive men and negative pregnant women. This number could demonstrate benefits of CHTC in ANC settings in identifying more HIV-positive men whose partners were negative than previous practice. CHTC is defined as when two partners are counseled, tested and receive their results together; in this way they can mutually disclose their HIV status with one another.

Characteristics of hospitals are shown in Table 1. Cross-sectional data were collected from pregnant women who came alone or with their partners at first visit, by interview using standard data collection forms. Pregnant women who came alone were offered routine ANC and encouraged to invite their partners for CHTC during the following visit. No incentives were provided for participation. A token amount of funding was provided to a nurse or a counselor who collected data (2 USD for data collection per case).

**Table 1:** **Characteristics of CHTC pilot implementing sites, 2009-10**

**Type of hospitals**	**Region**	**Number of new ANC cases during reporting period (Total n = 4524 cases)**	**% of pregnant women came with partners (2,089 cases)**	**% couples received pretest couples counseling (2,003 cases)**	**% couples accepted HIV testing (1,723 couples)**	**Number of counselors who provide CHTC (persons)**	**Type of CHTC**
Provincial Hospital	1. North	603	329 (54.6)	322 (97.9)	311 (96.6)	4	IC, CC, GE
	2. Northeast	761	119 (15.6)	118 (99.2)	117 (99.2)	4	IC, CC, GE
	3. Central	442	385 (86.9)	364 (94.5)	325 (89.3)	4	IC, CC, GE
	4. South	401	71 (17.7)	71 (100)	59 (83.1)	1	IC, CC, GE
	5. South	340	85 [25]	85 (100)	79 (92.9)	4	IC, CC
Community hospital	6. North	43	32 (74.4)	32 (100)	32 (100)	4	IC, CC
	7. North	136	43 (31.6)	43 (100)	43 (100)	5	IC, CC
	8. Northeast	199	78 (39.2)	78 (100)	75 (96.2)	3	IC, CC, GE
	9. Northeast	3	3 (100)	3 (100)	3 (100)	2	IC, CC
	10. Central	Not in database	Not in database	Not in database	Not in database	6	IC, CC
	11. South	346	108 (31.2)	107 (99.1)	47 (43.9)	3	IC, CC
	12. South	130	60 (46.2)	59 (98.3)	57 (96.6)	5	IC, CC
	13. South	142	81 (57)	76 (93.8)	71 (93.4)	2	IC, CC, GE
Health Promotion Center Hospital	14. North	410	284 (69.3)	269 (94.7)	150 (55.8)	3	IC, CC, GE
	15. Northeast	523	394 (75.3)	359 (91.1)	339 (94.4)	5	IC, CC, GE
	16. South	45	17 (37.8)	17 (100)	15 (88.2)	4	IC, CC

IC = individual counseling; CC = couples counseling; GE = group education and consent.

HIV counseling and testing data were collected at each woman’s first ANC visit, including types of pre- and post-test counseling, uptake of HIV testing by couples, reasons for not testing individually or as a couple, and HIV test results. Each hospital reported data from monthly paper-based monitoring forms for data entry at DOH in an electronic file using MS Access. Partners who did not return for posttest counseling within 3 months were defined as missing. Patient hospital numbers were recorded in the paper forms at the hospitals but were excluded from electronic transmissions to DOH. A couple code, linking separate records for patients and their partners, was used as an identifier in the transmissions to DOH and in the electronic database.

HIV enzyme-linked immunosorbent assays (ELISA) were performed for HIV-1 for those who consented to HIV testing. Each participant was asked individually whether they would prefer to receive HIV test results and posttest counseling alone or as a couple. HIV test results were given to pregnant women and partners at their next ANC visit. Individual counseling was provided to any participant upon request.

### Data analysis

Data from all participants were included in the analysis. Data were analyzed using STATA 11.0 (StataCorp., College Station, TX, USA) at the TUC office. We determined service uptake of individual or couples pre-test counseling, results of HIV testing among couples, barriers to testing, and percentage of HIV-infected women and partners referred to HIV care.

### Assessment of feasibility and acceptability of CHTC services

Following a minimum of 4 months of CHTC implementation, DOH and TUC staff provided monitoring and supervision visits at the pilot hospitals. The supervision team conducted in-depth interviews with hospital directors using a semi-structured interview guide to assess acceptability, support, and sustainability of CHTC services from an administrative/executive perspective. If the hospital director was not available, a hospital executive administrator (e.g. attending physician or chief of the ANC clinic) responsible for CHTC services was interviewed. There were three trained interviewers who alternately interviewed hospital executive administrators. Each in-depth interview session took about 30 minutes. All interviews were manually recorded as text by the interviewers. The data from in-depth interviews was organized by question and analyzed by identifying consistencies and differences of each responder. Comments from interviewees were summarized by categories.

### Assessment of staff responsible for CHTC services on feasibility, confidence, and experience in providing CHTC services

During site supervision visits, self-administered questionnaires were sent to staff responsible for CHTC services, asking about workload, confidence, CHTC practices, negative consequences from CHTC during the implementation period, and barriers to and recommendations for successful CHTC program implementation. We compiled the responses in Microsoft Excel 2010 (version 14). The data from self-administered questionnaires was analyzed and summarized.

### Ethics approval

This study was reviewed for human subjects considerations by the U.S. CDC and approved as research not involving human subjects. The Thailand MOPH determined this project to be a Program Evaluation, which does not require approval by the Thailand MOPH institutional review board.

## Results

### Characteristics of CHTC pilot sites

Among the 17 hospitals invited to participate in the CHTC trainings and pilot project implementation, 16 hospitals (94%) were able to implement CHTC services (Table 1). One community hospital was unable to implement CHTC services due to staffing limitations. One community hospital implemented CHTC services but did not collect and submit data to DOH; therefore, we report data from 15 (94%) of 16 pilot sites. The median number of counselors or nurses who provided CHTC in provincial hospitals, community hospitals, and health promotion center hospitals was 4 (range: 1–4), 3.5 (range: 2–6), and 4 (range: 3–5), respectively. All 16 hospitals also provided individual HIV counseling and testing for pregnant women and/or partners. Four (80%) of five provincial hospitals, two (25%) of eight community hospitals, and two (67%) of three health promotion center hospitals provided group HIV information before individual or couples pre-test HIV counseling (Table 1). There was significant variation in the proportion of pregnant women presenting with partners, and the proportion of couples accepting HIV testing, by hospital types and regions (p < 0.01) (data not shown).

### Uptake of pre-and post-test individual vs. CHTC

**Figure 2:**
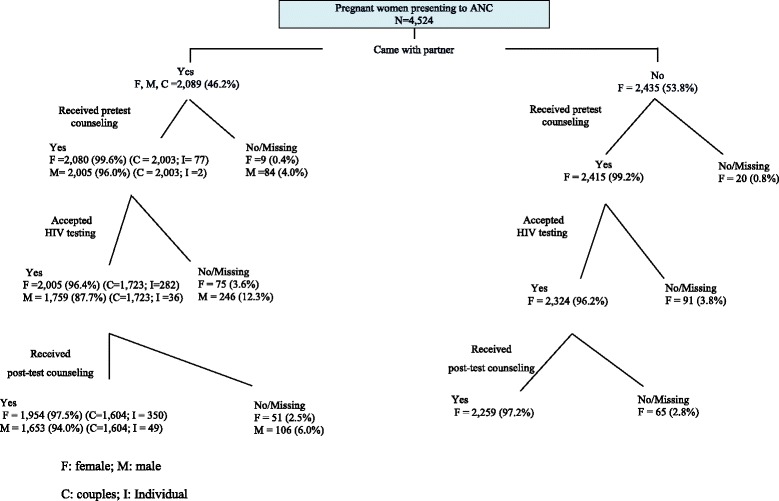
**Uptakes of couples HIV counseling and testing services among pregnant women and partners, Oct 2009 - April 2010.**

From October 2009 to April 2010, a total of 4, 524 women were enrolled from ANC clinics at the 15 hospitals (Figure 1). Of these, 2,089 (46%) women presented with their partners. Of these, 2,003 (96%) couples received pretest counseling (Figure 2), including 994 (50%) couples receiving couples pretest counseling, 864 (43%) receiving pre-test couples group education followed by couples consent, and 145 (7.3%) receiving individual risk assessment followed by couples counseling (data not shown). Among the 2,003 couples receiving pretest HIV counseling, 1,723 (86%) accepted HIV testing, and 1,604 (93%) of these returned for post-test counseling (Figure 2). Among couples who returned for post-test counseling, 1,443 (90%) received couples post-test counseling and the remainder received individual post-test counseling with or without mutual disclosure (data not shown). Cascades of pre-test and post-test counseling of pregnant women and their partners are shown in Figure 2.

Among 2,435 women who presented alone, 2,415 (99%) received pre-test HIV counseling. Of these, 1,341 (55%) received individual HIV counseling and testing, and 1,072 (44%) received group HIV education and consent. Of the 2,415 women receiving pre-test counseling, 2,324 (96%) accepted HIV testing. Two thousand two hundred and fifty-nine (97%) women returned for post-test HIV counseling.

### HIV-infection status among couples receiving CHTC and among pregnant women receiving individual counseling and testing

**Figure 3:**
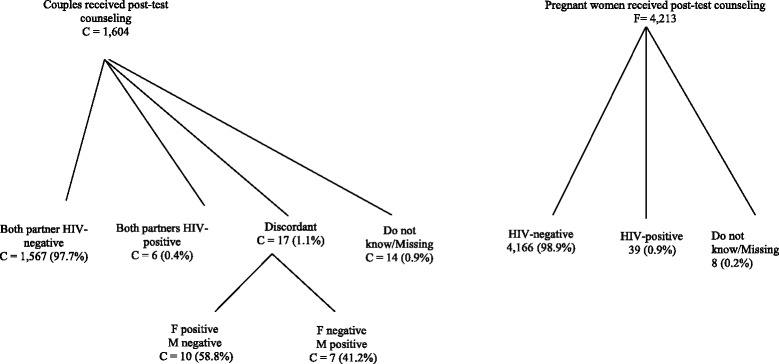
**HIV test results F: female; M: male; C: couples.**

Among 1,604 couples returning for post-test counseling, 1,567 (98%) were HIV concordant negative, 6 (0.4%) were HIV concordant positive, 17 (1%) were HIV discordant (7 male+/female- and 10 male-/female+), and 14 (0.9%) had no HIV test results (Figure 3).

Of 4,329 women who received HIV testing, 4,213 (97%) returned for post-test counseling. Of these, 4,166 (99%) were HIV negative and 39 (0.9%) were HIV positive. In this study, the overall HIV prevalence among men was 0.86% (15/1,746) and among pregnant women was 0.93% (40/4,298) (data not shown).

### Reasons for HIV test refusal among male partners

Of 2,005 men receiving pretest counseling, 1,759 (88%) men accepted HIV testing, including all partners of HIV-positive pregnant women. A higher proportion of men (891 (93%)) who received pre-test counseling at a provincial hospital accepted testing than men receiving pre-test counseling at either a community hospital (328 (82%)) or health promotion center hospital (504 (78%)) (Table 1). Among 246 men who refused HIV testing, 192 (78%) gave reasons for refusing testing. The main reasons were: did not want to test (115 (60%)); thought they had no risk (25 (13%)); wanted to test at hospitals near their residence (19 (10%)); fear of needles (17 (9%)); already knew HIV status (10 (5%)); and “other” (6 (3%)) (Data not shown).

### Feasibility and acceptability of CHTC services

Among the 15 hospitals, 10 (67%) hospital executive administrators participated in the in-depth interview including two hospital directors, four attending physicians, and four chief ANC nurses. Five hospital executive administrators were not available to participate in the interview on the days of the supervision visits. Median age was 43 years (range: 20–60 years) and 5 (50%) were male. Of these, 9 (90%) reported high acceptability for CHTC services due to perceived benefits for pregnant women, which included: early access to HIV prevention, treatment, and care; improved couples communication; and the ability to integrate the program with existing services. Nine interviewees (90%) thought that CHTC services could be integrated into routine services after the end of the pilot period. One interviewee reported “needing more data before making a conclusive assessment about the benefits of CHTC”, concerned that “some couples might have confidentiality concerns and may prefer individual HIV counseling and testing”.

Supportive systems for CHTC implementation available at hospitals included: written policies for CHTC (7/10); a CHTC working group (9/10); CHTC training (10/10), and CHTC campaigns at hospitals (8/10). Key barriers included: inadequate number of staff (5/10); staff being too busy (3/10); lack of tools (2/10), no clear defined leader for CHTC services on the team (2/10); not including provision of CHTC services in promotion criteria for staff (2/10); and no clear policy or standardized guidelines on how to provide CHTC in ANC settings (1/10). Concerns included potential negative consequences of CHTC for discordant couples, and additional staff workload.

Twenty-eight health care workers (HCWs) from the 15 hospitals participated in the survey including 17 nurses and 6 counseling nurses. Median age was 43 years (range: 33–54 years) and 26 (93%) were female. They reported between 2 and 65 clients per day in the clinic, with each HCW providing CHTC to up to 16 couples per day. More than 85% of HCWs reported that they were confident in providing post-test counseling for couples with HIV seroconcordant negative, seroconcordant positive, and serodiscordant results. Among 22 HCWs, 10 (45%) provided group pre-test CHTC and 6 (27%) provided group post-test CHTC. Of 14 HCWs who reported that they had experience in providing post-test counseling for discordant couples, 4 (29%) HCWs reported negative consequences of mutual disclosure of HIV results for some clients, such as separation or family conflict.

HCWs reported barriers for CHTC program implementation that included: high workload and limited number of staff trained in CHTC, leading to extended waiting times for clients; lack of clear national and hospital policies for CHTC implementation; a lack of standardized tools and materials to promote male participation in CHTC services at hospitals and in the community; partners of pregnant women refusing to participate in CHTC services; limited private space to provide CHTC services; and no waiting area for partners of pregnant women.

HCWs provided recommendations for improvement of CHCT program implementation, including: developing and disseminating clear CHTC national and hospital policies; free HIV testing support to partners of pregnant women; better support from program managers; adequate number of staff allocated for counseling; provision of private rooms for counseling and better space for group education; ongoing CHTC training and supervision for HCWs; development of a CHTC manual on how to organize CHTC services in hospitals with high numbers of new ANC cases that also have a limited number of counselors; and tools and materials to promote access of male partners to CHTC services and to use as guidance for new counselors. In addition, several HCWs interviewed suggested that CHTC uptake should be included as a national target indicator for maternal child health counseling.

## Discussion

This pilot project of a CHCT program at 15 hospitals in Thailand demonstrated that successful CHTC implementation in ANC setting is feasible. Uptake of CHTC services among male partners of pregnant women in ANC settings was high, as was acceptability among hospital staff and administration. Most hospitals participating in the program were able to successfully implement CHTC services. While this is the first report of a project of this type from Thailand, these findings are similar to those reported from sub-Saharan Africa and Cambodia [9-11]. Developing clear CHTC policies, having strong support from program managers, having a CHTC manual and materials, and training adequate numbers of HCWs to provide services were important elements contributing to successful program implementation. HCWs reported a high level of confidence in providing CHTC services, suggesting that the adapted training curriculum was sufficient to provide basic knowledge and skills for counselors.

Male participation in and uptake of CHTC services in this project was higher than that reported in African and Indian settings [10,22], although overall uptake of CHTC services in this project was still well below 50%. Possible reasons for the high rate of couples accepting HIV testing in our report may be due to one or more of several factors: first, CHTC in most pilot hospitals used opt-out techniques; second, the universal health coverage scheme in Thailand provided free HIV testing for pregnant women, partners of pregnant women, and other risk populations [18]; and third, Thailand promoted integration of CHTC in ANC settings with other existing maternal and child health (MCH) programs [23] in order to reduce stigma and discrimination. In this study, the proportion of couples accepting HIV testing was lower than 60% in one community hospital and one health promotion center hospital. Hospital staff reported that possible reasons for this low uptake included counselor issues (e.g., encouraging only partners of HIV-infected pregnant women or partners with risk behaviors to get tested), and health worker issues (e.g., workers were not aware of the health benefit of free HIV testing for partners of pregnant women). We do not have data on reasons for non-presentation of male partners at ANC, and it is possible that some men were not aware of the potential benefits of attending ANC with their partners due to a lack of promotional campaigns in some hospitals and communities, were not aware of availability of CHTC services in the ANC setting [23,24], were not available due to employment or residence in another province, were afraid to learn the results of HIV testing, had other socioeconomic factors or relationship issues affecting their decisions [24], or experienced unfriendly services for men at these CHTC service delivery sites [25]. Strategies that have been used in health facilities in Thailand and other countries to increase male involvement have included: making ANC services more male friendly (e.g., providing comfortable waiting space or fast-tracked registration for men); providing health education to change beliefs and attitudes; [10] and integration of ANC CHTC with other MCH programs such as thalassemia screening, syphilis screening, and parenting classes [8]. Additional strategies to attract men to ANC in Thailand should be instituted and evaluated; for example, in addition to those strategies noted above, engaging celebrities as role models [26] to promote CHTC services. In addition, for men who do not participate in CHTC services during ANC, CHTC should be offered to these men during the delivery or postpartum period where most men do present with their partners [1,8,25].

Loss to follow-up for men was also an issue in this project, possibly related to the fact that most participants needed to return for posttest results at the following clinical visit. The return rate for men may be improved by implementing same day HIV test results [27]. Feasibility and acceptability of using same day results among male partners of pregnant women in ANC settings should be examined.

Identifying new HIV cases is a cornerstone of HIV control, and identifying serodiscordant couples is critical for proper HIV prophylaxis, treatment and care, for women, men, and babies. In this study, a total of 13 HIV-positive men were identified during CHTC. Of these men, seven had HIV negative partners and would therefore not have been captured during standard ANC HIV-testing practices. Clearly, identifying new HIV cases is important in any venue. Identifying serodiscordant couples in which the male partner is HIV positive, is important to determine proper PMTCT interventions and identify women who may be in the window period of acute HIV infection. For HIV-infected persons, CHTC can also support more effective provision of ART and adherence [28], and can increase uptake and adherence of PMTCT [22], including early infant HIV diagnosis. Supporting couples counseling also allows couples the opportunity to share their vision related to family goals, and to make informed decisions about HIV and STI prevention and reproductive health, including family planning and contraception [1,29].

Although this pilot project demonstrated success, national adoption and scale up requires overcoming some substantial obstacles. Some HCWs in this pilot expressed concern about additional workload and negative consequences of CHTC, particularly for serodiscordant couples. Providing information to couples as a group, using videotape and/or in-person didactic counseling [30], was a common technique used in this project, particularly in tertiary care hospitals with high numbers of new ANC cases and limited staff. Studies from Africa have demonstrated varying associations between HIV diagnosis and intimate partner violence [25,26]. Limited information exists regarding these types of social outcomes of CHTC in Thailand [31], and more research is needed in this area to determine potential adverse consequences of positive test results, especially for discordant couples.

The interpretation of these results is subject to at least four limitations. First, the enrollment was a convenience sample. We did not collect data on the total number of pregnant women and their partners who came to the first ANC visit during the review period, or the number of those who were approached to participate in this project. Based on the national PMTCT intervention monitoring system that reported from participating hospitals during the same period of enrollment, it was estimated that the enrollment covered approximately 50% of total pregnant women at participating sites. Therefore, it is possible there was sampling bias, and this might lead to over or under estimation of uptake of couples counseling if expanded nationally. Second, missing data were reported and this may have led to over or under estimates of some of the findings. Third, there were variations in the proportion of men presenting at different ANC setting, and in the uptake of pre-and post-test CHTC by hospital type and region. These variations are likely to have implications for scale-up of this initiative and findings may need to be interpreted in specific regional and other contexts for optimal program implementation. Finally, we did not collect demographic information of participants and other information related to factors associated with acceptance of CHCT services. This information would be useful to help determine feasibility of national scale-up of this program, and is an area for future study.

Following implementation of this pilot CHCT program, revised Thailand PMTCT guidelines, released in October 2010 [18,19], recommended routine couples counseling and testing in ANC clinics. In 2013, CHTC programs in ANC settings were available in more than 70% of MOPH hospitals in all provinces in Thailand, but CHTC uptake was still low (22%) [13]. Moving forward, we recommend promoting involvement of male partners in ANC by providing and improving male-friendly services (e.g., providing free HIV testing, reducing waiting times, and providing waiting space for men), providing training for HCWs to increase couples counseling and testing skills, and support for CHTC implementation from hospital executives, all crucial for successful program implementation. Outcomes of couples counseling and testing during national implementation, and feasibility and acceptability of same-day test results for male partners should be studied in order to maximize program success.

## Conclusions

CHTC implemented in ANC settings helped identify more HIV-positive men whose partners were negative than previous practice, with high acceptability among HCWs. Major concerns were negative consequences of CHCT for discordant couples and additional workload of the program. Among couples who came to first ANC visit together, a high proportion received HIV counseling and testing. Strategies are needed to increase the number and proportion of male partners who access CHCT services. To implement CHCT, hospitals need clear policies, support from hospital leaders, trained and supported personnel, private space, training, tools, and materials. There exists an opportunity to identify new HIV cases, and prevent infection among children – both major aspects of a comprehensive HIV program, and achievable in Thailand.
